# Good Clinical Practices for the Management of Cervical Dystonia with BoNT-A: A Delphi-Based Approach from the Italian Expert Group

**DOI:** 10.3390/toxins18020079

**Published:** 2026-02-02

**Authors:** Roberto Eleopra, Marcello Esposito, Anna Rita Bentivoglio, Maria Concetta Altavista, Roberto Erro, Patrizia Maria Caglioni, Anna Castagna

**Affiliations:** 1 Parkinson and Movement Disorders Unit, Department of Clinical Neuroscience, Fondazione IRCCS Istituto Neurologico Carlo Besta, 20133 Milan, Italy; 2 Azienda Ospedaliera di Rilievo Nazionale (AORN) Antonio Cardarelli, 80131 Naples, Italy; 3 Neurology Unit, Fondazione Policlinico Universitario A. Gemelli IRCCS, 00168 Rome, Italy; 4 Neurology Unit, San Filippo Neri Hospital, ASL Roma 1, 00135 Rome, Italy; 5 Department of Medicine, Surgery and Dentistry “Scuola Medica Salernitana”, University of Salerno, 84081 Baronissi, Italy; 6 Ipsen, 20124 Milan, Italy; patrizia.caglioni@ipsen.com; 7 IRCCS Fondazione Don Carlo Gnocchi, ONLUS, 20162 Milan, Italy

**Keywords:** cervical dystonia, botulinum neurotoxin type A, good clinical practice, clinical management

## Abstract

Cervical dystonia (CD) is the most common adult-onset focal dystonia, with heterogeneous clinical presentation and significant functional impairment. Currently, no structured Italian good clinical practice documents specifically addressing CD have been published. Optimizing CD management requires expert-based recommendations to guide diagnosis, treatment, and follow-up. A two-round Delphi process was conducted, involving a scientific board of six neurologists with expertise in CD management and an external panel of 56 Italian experts (neurologists and physiatrists managing CD patients). Fifty-two statements were developed, discussed, and voted using a 5-point Likert scale, with consensus defined as ≥75% agreement (‘strongly agree’ or ‘somewhat agree’). In Round 1, 48 of 52 statements (92.4%) reached consensus; the four remaining statements were revised, and two were re-voted in Round 2, both achieving consensus. Final recommendations emphasize comprehensive patient assessment in multiple postural conditions; individualized botulinum neurotoxin type A (BoNT-A) dosing taking into account tonic and phasic components, pain, and dysphagia; the use of instrumental guidance; standardized outcome measures; and integration of physiotherapy and psychological support. This article provides structured good clinical practice recommendations for CD management and offers clinicians, especially those with limited experience, a practical framework to standardize care, optimize treatment, and improve patient outcomes.

## 1. Introduction

Cervical dystonia (CD) is a chronic neurological condition characterized by involuntary, sustained contractions of the cervical muscles. Clinically, it manifests with sustained or intermittent abnormal movements, inducing abnormal postures, tilting, twisting of the neck (in various combinations), and displacement of the head [1,2]. Common associated features include head tremor and neck pain [3,4,5]. The typical onset of CD usually occurs in adulthood, predominantly during the fifth and sixth decades of life, while early-onset cases (<28 years) are rare [6]. CD is the most common form of focal dystonia in adults, and diagnosis primarily depends on clinical evaluation, given the absence of a definitive laboratory test [7]. The pathophysiology of CD remains incompletely understood. Historically, basal ganglia dysfunction has been considered a key contributing factor in CD and other cases of focal dystonia. However, evidence suggests that dystonia arises from broader network dysfunction, implicating aberrant interactions between the basal ganglia and cerebellar motor circuits. This conceptual framework emphasizes dystonia as a disorder of impaired sensorimotor integration rather than a pathology confined to a single neural substrate [3]. Furthermore, recent studies have highlighted the significance of abnormalities in sensory processing and deficits in inhibitory control mechanisms in contributing to both the motor symptoms and the pain commonly reported by patients with CD. These impairments may contribute to the maladaptive sensorimotor integration underlying the disorder, further exacerbating symptom severity [8]. Additionally, the cerebellothalamo-cortical network appears to play a contributory role in the underlying pathophysiology of dystonia [9]. The burden of CD goes beyond its physical symptoms and manifestation, resulting in stigma and impaired quality of life and affecting the psychological well-being of patients. Furthermore, the debilitating nature of CD symptoms often results in reduced physical activity and social participation, leading to further psychological distress [10]. Although scientific evidence on benefit is still scarce, in clinical practice, potential effective treatment strategies for CD include both pharmacological and non-pharmacological approaches, such as sensori-motor retraining exercises inspired by motor learning paradigm, passive or active mobilization techniques, stretching of dystonic muscles, relaxation, and electrotherapy (e.g., electromyography (EMG)) biofeedback or transcutaneous electrical nerve stimulation (TENS) [11,12]. Among pharmacological treatments, botulinum neurotoxin represents the first-line treatment for patients with CD [11,12,13,14,15].

Additionally, deep brain stimulation (DBS) demonstrated promising results, effective in improving dystonia and patient outcomes [16]. Despite these advances, several unmet needs persist in the management of cervical dystonia. Notably, there are significant gaps in both diagnosis and treatment, reflecting a lack of standardized care pathways and comprehensive treatment protocols. A prominent issue is the considerable variability in treatment responses and outcomes among patients, which complicates the development of a unified clinical practice protocol. Moreover, due to the lack of a diagnostic test, cervical dystonia diagnosis is based on clinical examination, history, clinical features, and phenomenology, and is therefore subjective [7,17].

To our knowledge, no national Italian guidelines or formal good clinical practice documents specific to the management of CD have been published. Here, we present a Delphi consensus study aimed to provide principles of good clinical practices for the evaluation, management, and treatment of CD. The absence of Italian-specific guidelines on this condition was the primary motivation for the development of this work, which is intended to serve as a practical reference for clinicians, particularly those with limited experience in botulinum toxin therapy, by providing structured, expert-based recommendations across key domains of assessment and treatment. By drawing on the expertise of experienced clinicians, this study aims to streamline clinical decision-making, improve treatment effectiveness, and deliver evidence-based recommendations to enhance patient outcomes. Consensus findings will contribute to a more structured and efficient approach to cervical dystonia management, ultimately advancing the standard of care for individuals with CD.

## 2. Results

**Figure 1:**
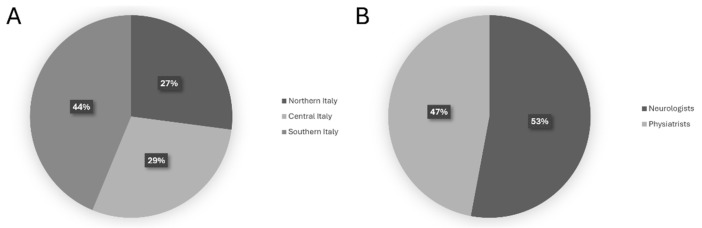
(**A**) Geographical distribution and (**B**) specializations of the expert panel.

The methodology and the composition of both the scientific board committee and the expert panel are described in detail in the Materials and Methods Section 5. On 9 January 2025, Round 1 (R1) was sent out for voting and finalized on 21 January 2025, and the 56 external panelists expressed their consensus on the 52 statements. All members of the panel were clinically experienced, with more than 5 years dedicated to the management and treatment of cervical dystonia using botulinum toxin type A (BoNT-A), representing a broad range of clinical expertise and practice setting, and regional distribution, with experts from Northern (27%), Central (29%), and Southern (44%) Italy (Figure 1A). It included both neurologists (53%) and physiatrists (47%) (Figure 1B), as well as professionals from hospital-based settings (76%) and academic institutions (24%), ensuring a heterogeneous and balanced perspective on the assessment and management of cervical dystonia.

The return rate during R1 was 100%. The list of the statements submitted for the first e-Delphi round and their respective consensus are reported in Table 1. Out of 52 statements, only four (7.6%) statements did not reach consensus:

32. *When facing an anterocollis/antecaput, it is necessary to perform dynamic cervical X-ray (e.g., with patients performing head flexion and extension) to identify involvement of deep muscles (long head–neck, e.g., longus collis, longus capitiis).*

41. *A dedicated follow-up for pain, regardless of the clinical changes in dystonic postures.*

43. *In case of partial or no response, a neurophysiological test (Extensor Digitorum Brevis) should be performed to quantify and verify the response to the toxin (induced neuromuscular blockade).*

48. *In post-treatment follow-up, in selected cases where clinical efficacy is very short, it is preferable to repeat the treatment after 2 months following the recurrence of symptoms.*

**Table 1:** Statements on cervical dystonia submitted for the first e-Delphi round (R1). Gray rows include statements that did not reach consensus.

**Domain: Conditions for Clinical Evaluation of the Patient**	**Consensus (%)**
1. It is necessary to evaluate patients while sitting at rest with their eyes open.	96.2%
2. It is necessary to evaluate patients while sitting at rest with their eyes closed.	90.4%
3. It is necessary to evaluate patients while seated at rest in the Mingazzini I position, with their eyes open.	80.8%
4. It is necessary to evaluate patients while seated at rest in the Mingazzini I position, with their eyes closed.	80.8%
5. It is necessary to evaluate the voluntary movement of the head across different planes while the patient is seated.	98.0%
6. It is necessary to evaluate the voluntary movement of the head across different planes while the patient is standing.	96.2%
7. It is necessary to evaluate the patient standing with the eyes open.	92.3%
8. It is necessary to evaluate the patient standing with the eyes closed.	84.6%
9. It is necessary to evaluate the patient while walking.	100%
10. It is necessary to evaluate the prevalent pattern of cervical dystonia.	98.1%
11. It is necessary to evaluate the severity of cervical dystonia.	100%
**Domain: Additional Evaluation Parameters Beyond the Predominant Pattern for Injection Target Selection**	
12. The tonic (postural) component of dystonia must be considered when choosing treatment targets.	100%
13. The phasic (mobile) component of dystonia must be considered when choosing treatment targets.	98.1%
14. Tremor must be considered when choosing treatment targets.	98.1%
15. Shoulder positioning must be considered when choosing treatment targets.	100%
16. Muscle trophism is an element to consider when choosing possible dosage targets.	98.1%
17. Pain is an element to consider when choosing targets.	86.5%
**Domain: Additional evaluation parameters to the severity of cervical dystonia for dosage selection**	
18. The tonic (postural) component of dystonia must be considered when choosing treatment dosages.	92.3%
19. The phasic (mobile) component of dystonia must be considered when choosing treatment dosages.	90.3%
20. Tremor must be considered when choosing treatment dosages.	86.5%
21. Shoulder positioning must be considered when choosing treatment dosages.	82.7%
22. Muscle trophism is an element to consider when choosing possible dosages.	92.3%
23. Pain is an element to consider when choosing treatment dosages.	86.5%
24. Dysphagia must be considered when choosing treatment dosages.	90.4%
25. Muscle trophism is an element to consider when choosing dosage.	94.2%
**Domain: Treatment with BoNT-A**	
26. At first inoculation, it is recommended to treat only the prevailing dystonic pattern.	96.2%
27. To optimize treatment, it is recommended to use instrumental guidance.	96.1%
28. To improve the localization of injection targets, it is preferable to use ultrasound guidance.	92.3%
29. To improve the identification of active targets, it is preferable to use EMG guidance.	94.2%
30. To optimize treatment in complex cases, it is recommended to use dual guidance.	92.3%
31. To optimize treatment in complex cases, it is recommended to perform a poly-EMG to identify the dystonic muscles to be treated.	90.4%
32. When facing an anterocollis/antecaput, it is necessary to perform dynamic cervical X-ray (e.g., with patients performing head flexion and extension) to identify involvement of deep muscles (long head–neck, e.g., longus collis, longus capitiis).	*42.3%*
33. When choosing the treatment, it is necessary to consider the patient’s expectations and adequately define the expected goals of the treatment with the patient and caregiver.	98.1%
34. In cases of head tremor, treatment of muscle groups bilaterally is necessary.	84.6%
35. Before starting treatment, it is appropriate to film the patient and the presence of abnormal postures.	94.2%
**Domain: Follow-up**	
36. When assessing post-treatment follow-up, it is recommended to use the Toronto Western Torticollis Rating Scale (TWTRS).	90.4%
37. When evaluating post-treatment follow-up, it is recommended to use the Tsui scale.	84.7%
38. When evaluating post-treatment follow-up, it is necessary to film the patient.	90.4%
39. It is necessary to perform at least four cycles of treatment before defining clinical nonresponse.	88.4%
40. In the poorly responsive or nonresponsive dystonic patient, a poly-EMG should be performed to identify the dystonic muscles to be treated.	92.3%
41. A dedicated follow-up for pain, regardless of the clinical changes in dystonic postures.	*71.2%*
42. In case of partial or no response, it is necessary to reevaluate the patient clinically and instrumentally (poly-EMG).	94.3%
43. In case of partial or no response, a neurophysiological test (Extensor Digitorum Brevis) should be performed to quantify and verify the response to the toxin (induced neuromuscular blockade).	*69.3%*
44. In evaluating the response to treatment, it is advisable to assess the psychological state of the patient.	94.2%
45. When evaluating treatment response, it is necessary to consider the patient’s expectations/adequately define the expected goals with the patient and caregiver.	100%
**Domain: Timing**	
46. After the first treatment, it is necessary to evaluate the effectiveness of the treatment between 4 and 6 weeks after inoculation.	92.3%
47. In post-treatment follow-up, it is recommended to wait at least 3 months before repeating inoculations with toxin.	98.1%
48. In post-treatment follow-up, in selected cases where clinical efficacy is very short, it is preferable to repeat the treatment after 2 months following the recurrence of symptoms.	*63.5%*
49. After each injection cycle, adverse events should be considered in order to optimize the choice of muscles to treat and the doses to be used.	100%
**Domain: Additional post-inoculation treatments**	
50. It is advisable to combine physiotherapy with toxin treatment.	90.2%
51. It is advisable to combine self-rehabilitation with toxin treatment.	84.7%
52. It is advisable to combine psychological treatment with toxin treatment in patients with emotional distress.	90.3%

The four statements that did not reach consensus underwent a new revision. After an in-person meeting held by the scientific board, only statement 41 and statement 43 have been rephrased and approved, as shown in Table 2. The board decided to eliminate statements 32 and 48 from the second round of voting. On 10th February 2025, Round 2 (R2) was sent out, and the results were collected on 23rd February 2025, and 41 panelists out of 56 completed R2 (return rate: 73%). Both statements in R2 reached consensus (Table 2).

## 3. Discussion

Cervical dystonia (CD) is a motor disorder characterized by abnormal postures and often repetitive movements caused by involuntary muscle contractions. CD is often accompanied by pain and tremors, with an estimated prevalence of 20–4100 cases/million. CD represents a large burden for affected patients with a substantial impact on their quality of life [18]. In this Delphi study, we aimed to establish and provide an indication for good clinical practice for assessment, treatment, and management of CD among Italian specialists. During the first round of voting, most of the statements achieved a large consensus among panelists. Only in three domains (treatment with BoNT-A, follow-up and timing) did some statements not initially reach consensus, which was subsequently achieved after R2 revision and voting. The high level of agreement observed in this Delphi process may be explained by the relative homogeneity of therapeutic approaches to cervical dystonia described in the literature. As the expert panel was composed of experts who have directly contributed to the body of published evidence, it is reasonable that their views aligned closely with established practices, thereby resulting in the elevated degree of consensus across the statements. In the conditions for clinical evaluation of the patient, a strong agreement was reached on the importance of assessing patients in various postural conditions, including sitting at rest (96.2% eyes open, 90.4% eyes closed), standing (92.3% eyes open, 84.6% eyes closed), and walking (100%). These results align with previous studies emphasizing the need for dynamic assessments in CD to capture the full spectrum of dystonic movements and compensatory mechanisms [19]. Notably, the consensus also supports the evaluation of voluntary head movements in both seated (98.0%) and standing (96.2%) position, further highlighting the importance of functional assessments in guiding treatment decisions. Analysis of R1 indicated that the Mingazzini I position is crucial as part of the evaluation process, with 80.8% agreement for both eyes open and closed. Although not traditionally emphasized in the assessment of cervical dystonia, evaluating patients in this position may enhance the identification of abnormal muscle activation patterns. Regarding treatment selection, there was unanimous agreement (100%) that both the tonic (postural) and phasic (mobile) components of dystonia should be considered when determining the appropriate dosages of BoNT-A. These results are in line with prior literature suggesting that an individualized approach, considering not only predominant movement patterns but also tremors (98.1%), shoulder positioning (100%), and muscle trophism (98.1%), is essential for optimizing therapeutic outcomes [20]. Additionally, pain was recognized as a relevant factor in dosage selection (86.5%) (Table 1). This supports existing evidence that pain significantly impacts quality of life in CD patients and should be an integral part of treatment planning [21].

Regarding dosage selection, there was strong agreement on the importance of considering both the tonic (92.3%) and phasic (90.3%) components of dystonia, reinforcing the need for an individualized approach that accounts for static and dynamic movement patterns [4]. Similarly, tremors (86.5%), shoulder positioning (82.7%), and muscle trophism (94.2%) were identified as fundamental in dosage determination, supporting the idea that CD should be assessed beyond simple severity scales, integrating functional and anatomical considerations. Additionally, the high consensus on pain (86.5%) and dysphagia (90.4%) as dosage-determining factors, either limiting or increasing, is consistent with prior research emphasizing the impact of non-motor symptoms in CD, which can significantly affect treatment response and patient quality of life [22]. In the domain of BoNT-A treatment, expert consensus strongly supports treating only the prevailing dystonic pattern during the first injection (96.2%), further supporting the importance of avoiding improper selection of active muscles and injections of muscles that are not responsible for specific patterns [23]. Instrumental guidance, particularly ultrasound (92.3%) and EMG (94.2%), was also highly recommended to improve injection accuracy and optimize efficacy, in line with growing evidence that these techniques enhance localization of dystonic muscles and reduce treatment variability [4,24]. Interestingly, the statement regarding the use of dynamic cervical spine X-ray to identify deep muscle involvement in anterocollis was not widely endorsed and did not achieve consensus during Round 1. Given the topic’s complexity, the rarity of this particular CD phenotype and the lack of agreement, the scientific board decided to remove this statement from the consensus. The panel was unable to reformulate the item in a way that clearly and accurately conveyed the underlying concept without risking ambiguity or potential misinterpretation. This decision reflects a deliberate methodological choice to avoid retaining statements that, despite potential clinical relevance, could be misleading if not precisely expressed. Similarly, the lack of agreement on statement 48 likely reflects concerns of responders regarding safety, feasibility, and the absence of clear evidence to support this strategy. Finally, patient-centered care was emphasized, with 98.1% of experts recommending that treatment expectations be discussed with patients and caregivers prior to initiation. This result aligns with the evolving approach toward shared decision-making in cervical dystonia and other movement disorders, aiming to establish realistic treatment goals and ensure that patients’ expectations are appropriately managed [25]. Furthermore, the high consensus on pre-treatment video recording (94.2%) reflects the need for objective documentation of baseline dystonic postures, helping in treatment planning and longitudinal outcome assessment. This large consensus reflects the current literature, which supports the use of videos and other innovative techniques for the correct evaluation of abnormal postures in patients with cervical dystonia [26,27].

There was a strong consensus on the importance of standardized outcome measures for post-treatment assessment. The external panel recommended the Toronto Western Torticollis Rating Scale (TWTRS) and Tsui scale, reflecting their widespread use in clinical practice and research for assessing disease severity and treatment response [28,29,30]. A notable agreement was reached on the necessity of at least four treatment cycles before defining clinical non-response, ensuring that patients receive an adequate therapeutic trial before considering alternative strategies. Interestingly, this might be in contrast with the definition suggested by other authors of clinical non-response. Some authors reported that two, four, or five injection cycles were necessary to define secondary non-response. Conversely, some definitions describe secondary non-response as an “unsatisfactory therapeutic response to two consecutive injection cycles, in patients who have previously experienced at least two successful treatment cycles” [31,32]. In clinical practice, this indication should be applied flexibly, balancing the need for adequate optimization with real-world constraints. Before defining non-response, clinicians should consider dose adjustments, muscle re-selection, the systematic use of instrumental guidance, and re-evaluation of the dystonic phenotype. In selected cases, earlier decision-making may be warranted due to adverse events, functional urgency, or patient preference.

Furthermore, in poorly responsive or nonresponsive patients, poly-EMG (92.3%) was strongly endorsed to refine muscle targeting, supporting prior evidence that this technique, although invasive, might allow for proper muscle target selection and enhance treatment effectiveness in selected cases [33]. Additionally, psychological assessment (94.2%) and discussing treatment expectations with patients and caregivers (100%) were identified as essential. Current literature supports the importance of psychological assessment of patients since people with dystonia can experience high levels of stigma and psychological distress, which might influence self-perception about the efficacy of BoNT-A treatment [34].

Regarding the timing domain, the external panel agreed that the optimal time for initial treatment evaluation is 4 to 6 weeks post-injection, aligning with the peak clinical effects of BoNT-A reported in the literature [25]. Additionally, a strong consensus supports maintaining a minimum 3-month interval between treatments. An important consideration was assessing adverse events after each injection cycle, emphasizing the need for ongoing risk–benefit evaluation and refinement of treatment parameters. Finally, as reported by other studies, the external panel provided strong consensus regarding the importance of physiotherapy and self-rehabilitation, despite the current knowledge gap, along with BoNT-A treatment even if robust evidence is still lacking and further research in this field is mandatory [35,36]. While these recommendations are supported by clinical experience and emerging literature, the available evidence remains heterogeneous, and these statements should be interpreted as expert indications rather than high-level evidence-based recommendations.

*Limitations of the Study*

This study has some limitations that should be acknowledged. Although expert consensus represents a valuable methodological approach in areas where high-quality evidence is limited or heterogeneous, it does not equate to high-level evidence and should be interpreted within this context. The recommendations presented herein reflect expert clinical experience and agreement rather than graded evidence-based guidelines. In addition, the national scope of the panel may limit the generalizability of these findings to other healthcare systems, and the composition of the panel may reflect prevailing specialist practices. Methodological rigor, including anonymous voting, predefined agreement thresholds, and iterative revision, was applied to preserve independence and mitigate potential bias. The entire Delphi process, including study design, data collection, analysis, and manuscript preparation, was organized and managed by an independent third-party agency to ensure scientific autonomy and minimize potential bias. Finally, patient representatives were not directly involved in the Delphi process, which should be considered given the emphasis on patient expectations, quality of life, and shared decision-making.

## 4. Conclusions

To date, there are no national Italian recommendations or structured documents outlining best clinical practices for the assessment and treatment of cervical dystonia. This lack of formal guidance represents a significant gap in clinical support, especially given the complexity and variability of the condition. This Delphi consensus was developed to address the existing gap in guidance and highlights key recommendations for optimizing the assessment, treatment, and follow-up of cervical dystonia (CD), emphasizing the necessity of a standardized yet individualized approach. The findings underscore the importance of comprehensive clinical evaluations, targeted BoNT-A injections, structured follow-up protocols, and the integration of adjunct therapies such as physiotherapy and psychological support. These indications are intended to be readily applicable in routine clinical practice, supporting structured patient assessment and standardized follow-up.

By implementing these expert-derived good clinical practices, clinicians can enhance treatment accuracy, improve patient outcomes, and adopt a more holistic approach to CD management. The expected impact on clinical practice is substantial, as the consensus promotes greater standardization, informed decision-making, and personalized patient care. In addition to supporting clinical decision-making in complex or atypical cases, this work aims to serve as a practical reference for healthcare professionals, particularly those new to managing cervical dystonia, fostering a more standardized, evidence-based approach to patient care. Future research should focus on validating these indications for good clinical practice through real-world clinical implementation, including structured monitoring pathways and multidisciplinary care models. Moreover, these findings may serve as a foundation for future national or international guideline development and inform the design of prospective observational studies and clinical trials aimed at optimizing the management of cervical dystonia with botulinum neurotoxin type A.

## 5. Materials and Methods

This study was conducted using the Delphi method to achieve consensus on good clinical practice in managing CD. An independent service provider ensured participant anonymity and minimized potential biases, further enhancing the validity and reliability of the consensus. The Delphi method is structured to facilitate expert discussions on complex topics [37,38]. It is widely used across various disciplines, particularly healthcare, to build consensus in areas where data are limited, guidelines are incomplete, or knowledge remains uncertain [39,40]. This method operates through three key stages: selecting a panel of experts based on their recognized international and/or national clinical expertise, significant scientific contributions through publications and research, active involvement in professional societies, and experience in peer-reviewed work (efforts were made to ensure a balanced representation of professionals with diverse perspectives within the field); developing structured surveys; and conducting iterative rounds to refine responses and achieve consensus. [40] The iterative process progressively narrows perspectives while maintaining consensus through three core principles: anonymity, controlled feedback, and statistical aggregation of group responses [41]. Our study was designed and performed between January 2025 and March 2025. A scientific board comprising 6 Key Opinion Leaders (KOLs) in neurology with expertise in CD management was established based on their clinical expertise in CD management. Three in-person meetings were held to define and refine seven key domains: conditions for clinical evaluation of the patient, additional evaluation parameters beyond the predominant pattern for injection target selection, additional evaluation parameters to the severity of cervical dystonia for dosage selection, treatment with BoNT-A, follow-up, timing, and additional post-injection treatments. During the first round (R1), 52 statements were formulated and distributed to an external panel of 56 Italian expert neurologists and rehabilitation medicine specialists with expertise in CD via an electronic platform. Panelists were asked to evaluate each statement and provide their responses following the Delphi methodology. Agreement or disagreement was rated individually and anonymously using a five point Likert scale (1 = strongly disagree, 2 = somewhat disagree, 3 = neither agree nor disagree, 4 = somewhat agree, 5 = strongly agree). Participants who rated a statement below 5/5 were required to provide a written explanation for their disagreement. Additionally, they could submit comments on each item, which were carefully reviewed and incorporated into the second round (R2) of the questionnaire. Consensus was defined as ≥75% of respondents selecting either ‘strongly agree’ or ‘somewhat agree.’ Statements that did not reach consensus in R1 were revised and included in the R2 questionnaire. Panelists were then invited to reassess and vote on the updated statements. In the final stage, a comprehensive data analysis was conducted, culminating in developing the final Delphi report under the guidance of a methodology expert.

## Figures and Tables

**Table 2:** Amended statements voted during the second e-Delphi round (R2).

Amended Statements	Consensus (%)
41.1 In post-inoculation, it is necessary to assess the pain symptom response independently of the dystonic motor response.	80.0%
43.1 To assess immune resistance to botulinum toxin, after repeated treatments with appropriate doses and targets with partial or no clinical response, it is recommended to perform neurophysiological testing (e.g., EDB Test).	92.5%

## Data Availability

The original contributions presented in this study are included in the article. Further inquiries can be directed to the corresponding author(s).

## Abbreviations

The following abbreviations are used in this manuscript:

CD	Cervical Dystonia
BoNT-A	Botulinum Neurotoxin type A
DBS	Deep Brain Stimulation
EMG	Electromyography
R1	Round 1
R2	Round 2
TENS	Electrical Nerve Stimulation
